# Supplementary material for: Implicit motor imagery performance is impaired in people with chronic, but not acute, neck pain
Source: PeerJ. 2020 Feb 14;8:e8553. doi: 10.7717/peerj.8553 (PMC7025709; doi:10.7717/peerj.8553)
Supplement: Supplemental Information 1 [file peerj-08-8553-s001.docx]

Supplemental File

**Aim 2:** The effect of side of neck pain on judgements to left turning and right turning neck images

**Response time:**

| **Comparisons** | **Mean difference** | **95% CI** | **p-value** |
| --- | --- | --- | --- |
| no pain – left-sided pain* | -132.643 | -245.439—19.847 | 0.021* |
| no pain – right-sided pain | -13.524 | -130.566-103.518 | 0.821 |
| no pain – bilateral pain* | -128.292 | -231.348—25.236 | 0.015* |
| left-sided pain – right-sided pain | 119.119 | -38.110-276.348 | 0.137 |
| left-sided pain – bilateral pain | 4.351 | -142.764-151.466 | 0.954 |
| right-sided pain – bilateral pain | -114.768 | -265.163-35.627 | 0.135 |

**Table 1**. Post-hoc response time comparisons for Side of Pain to judge a left-turning and right-turning neck image (*denotes significant between group differences).

Selecting only those with lateralised neck pain (left vs right):

There was a main within-subjects effect of Direction of Image Head Rotation (F_1,150_= 6.94, p=0.009, partial η^2^ =0.044) and no main between-subjects effect of Side of Pain (F_1,150_= 2.09, p=0.15, partial η^2^ =0.014) and no Direction of Image Head Rotation x Side of Pain interaction (F_1,150_= 3.66, p=0.058, partial η^2^ =0.024). People were faster at identifying a right-turning neck image than a left-turning neck image (mean difference: 49.61, 95% CI: 12.40-86.82, p=0.009).

**Sensitivity analysis: Pain evoked by neck movement**

The effect of neck pain evoked by side-specific movement on responses to left turning and right turning neck images.

Methods

To determine the *effect of neck pain evoked by side-specific movement* (i.e., neck pain evoked by neck rotation to the left, right or both; *Aim 3*) on left-turning and right-turning *neck* images, accuracy and response time were separately investigated using a 2 (within-subjects main effect of Side of Head Turn in Image: left-sided turning images and right-sided turning images) by 4 (between-subjects main effect of Movement-Evoked Neck Pain: no pain, left side, right side, bilateral) repeated measures ANOVA. To specifically evaluate the effect of lateralised neck pain during movement on performance (accuracy and response time), repeated measures ANOVAs were completed comparing only those with lateralised neck pain on movement (left-side versus right-side) for performance on left-sided turning and right-sided turning neck images

Results

**Overall sample – Accuracy:**

There was a main within-subject effect of Direction of Image Head Rotation (F_1,1271_= 26.51, p<0.001, partial η^2^ =0.020) and a main between-subject effect of Movement-Evoked Neck Pain (F_3,1271_= 9.163, p<0.001, partial η^2^ =0.021), but no Direction of Image Head Rotation x Movement-Evoked Neck Pain interaction (F_3,1271_= 0.094, p=0.963, partial η^2^ =0.000). Post-hoc analyses revealed that people were more accurate at identifying right-turning images than left-turning images (p<0.001) and people with no directional pain were more accurate than people with bi-directional pain (p<0.001; see Supplementary Figure 1A).

**Overall sample – Response time:**

There was a main within-subjects effect of Direction of Image Head Rotation (F_1,1273_= 11.37, p=0.001, partial η^2^ =0.009) and a main between-subjects effect of Movement-Evoked Neck Pain (F_3,1273_= 5.60, p=0.001, partial η^2^ =0.013), but no Direction of Image Head Rotation x Movement-Evoked Neck Pain interaction (F_3,1273_= 0.71, p=0.55, partial η^2^ =0.002). Post-hoc analyses revealed that people were faster at responding to a right-turning neck image than a left-turning neck image (mean difference= -47.69ms, 95% CI= -75.4ms to -19.9ms, p=0.001) and that people with no directional pain were faster than people with bi-directional pain (mean difference = -143.7ms, 95%CI -236.9ms to -50.5ms, p=0.015) and people with pain evoked by left-sided movement (mean difference = -181.0ms, 95%CI -330.4ms to -31.5ms, p=0.018), and approached significance for people with pain evoked by right-sided movement (mean difference = -149.7ms, 95%CI -300.9ms to 1.5ms, p = 0.052; see Supplementary Figure 1B).

| **Comparisons** | **Mean difference** | **95% CI** | **p-value** |
| --- | --- | --- | --- |
| no directional pain – left-sided directional pain* | -180.972 | -330.442—31.501 | 0.018* |
| no directional pain – right-sided directional pain | -149.701 | -300.873-1.471 | 0.052 |
| no directional pain – bilateral directional pain* | -143.669 | -236.878—50.459 | 0.003* |
| left-sided directional pain – right-sided directional pain | 31.271 | -177.234-239.775 | 0.769 |
| left-sided directional pain – bilateral directional pain | 37.303 | -133.897-208.502 | 0.669 |
| right-sided directional pain – bilateral directional pain | 6.032 | -166.655-178.719 | 0.945 |

**Table 2**. Post-hoc response time comparisons for Movement-Evoked Neck Pain to judge a left- and right-turning neck image (*denotes significant between group differences).

**Selecting only those with lateralised movement evoked neck pain (left vs right):**

Accuracy:

There was a main within-subject effect of Direction of Image Head Rotation (F_1,83_= 5.98, p=0.017, partial η^2^ =0.067) but no between-subject effect of Movement-Evoked Neck Pain (F_1,83_= 0.05, p=0.83, partial η^2^ =0.001), and no Direction of Image Head Rotation x Movement-Evoked Neck Pain interaction (F_1,83_= 0.12, p=0.731, partial η^2^ =0.001; see Supplementary Figure 2A).

Reaction time:

There was no main within-subjects effect of Direction of Image Head Rotation (F_1,83_ = 3.23, p=0.076, partial η^2^=0.037) and no main between-subjects effect of Movement-Evoked Neck Pain (F_1,83_ =0.08, p=0.779, partial η^2^=0.001), and no Direction of Image Head Rotation x Movement-Evoked Neck Pain interaction (F_1,83_ =1.87, p0.175, partial η^2^=0.022; see Supplementary Figure 2B).

Summary**:** People were faster and more accurate in identifying a right-turning neck image than a left-turning neck image, regardless of whether or not they experienced movement-evoked neck pain. There were no clear side-specific effects on performance – only differences based on location of movement-induced pain were found. Consistent with Aim 2, people with bi-directional movement-evoked neck pain were most affected – they were slower and less accurate at neck left/right judgements than those with no directional pain – and people with pain evoked by left neck rotation were slower than those with no directional pain. Similarly for unilateral pain, the side of movement-evoked pain did not influence performance: people with pain evoked by left neck rotation were no slower or less accurate at identifying images of left neck rotation than those with pain evoked by right neck rotation (and vice versa).
