# Supplementary figures and images for: Implicit motor imagery performance is impaired in people with chronic, but not acute, neck pain
Source: PeerJ. 2020 Feb 14;8:e8553. doi: 10.7717/peerj.8553 (PMC7025709; doi:10.7717/peerj.8553)

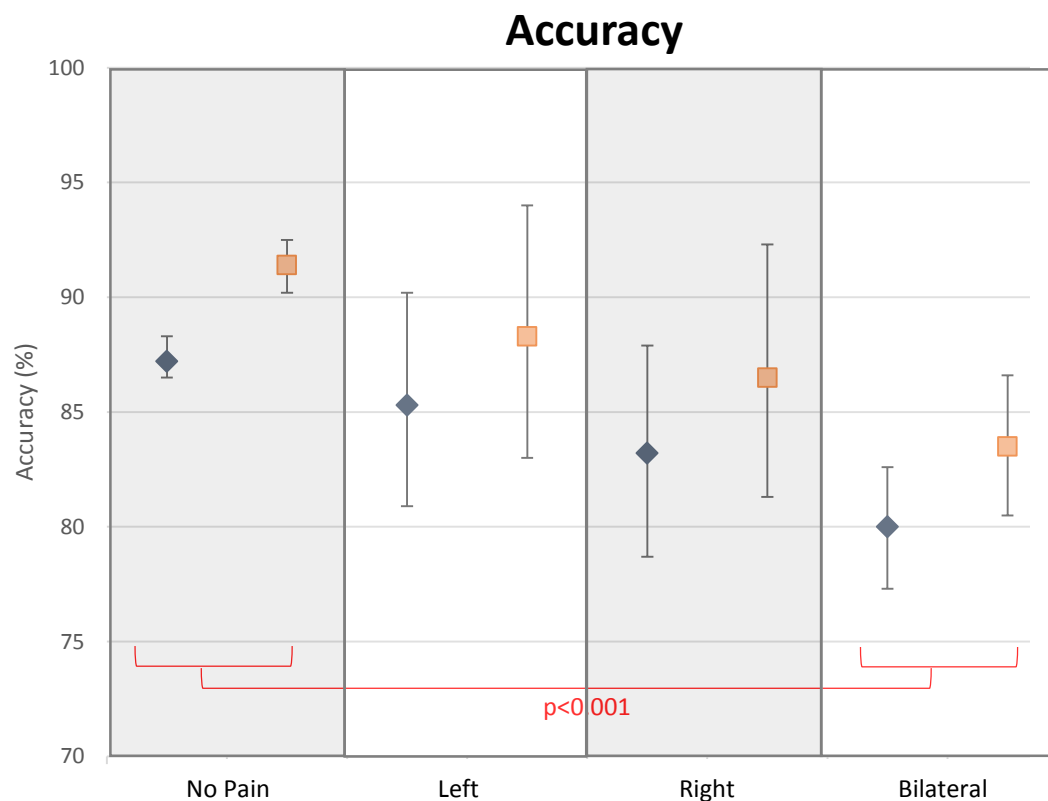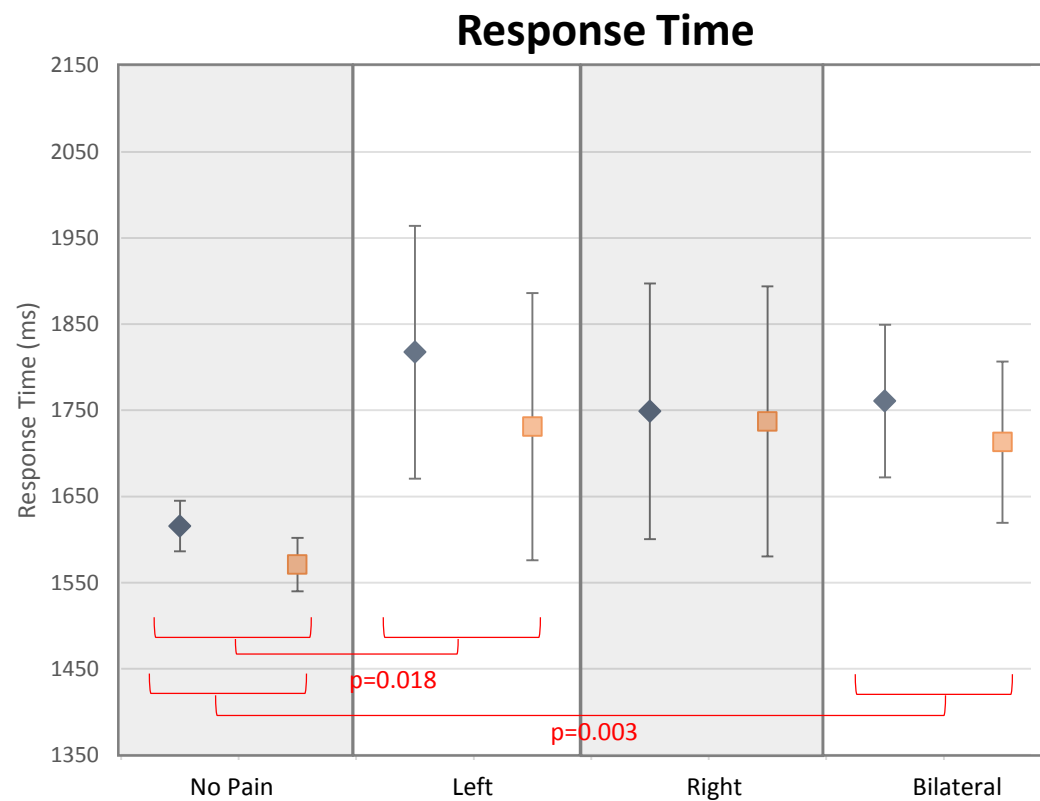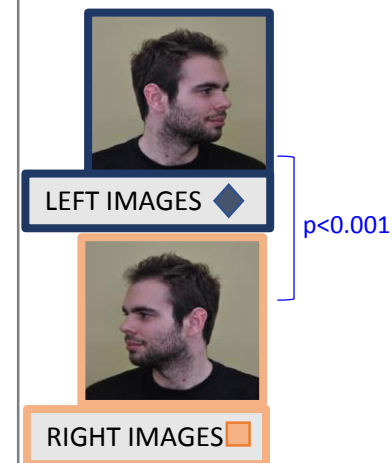

**A**

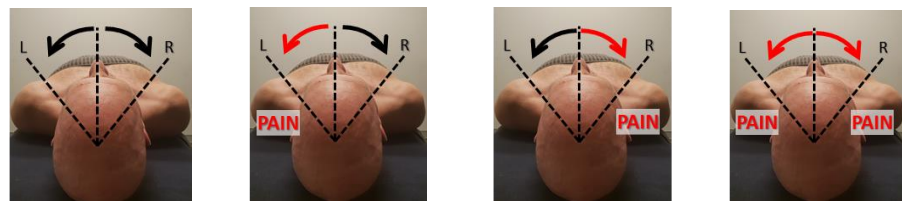

**B**

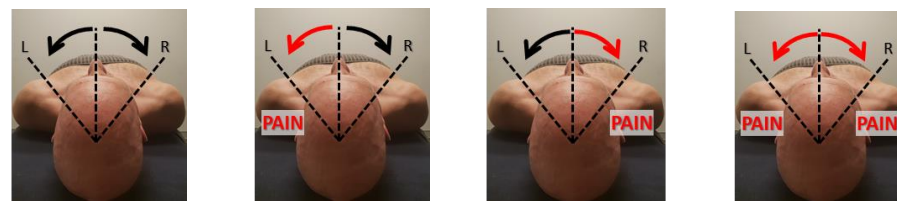

Direction of head turn associated with pain

Supplement: Supplemental Information 2 — (A) Accuracy. (B) Response time. Those with bilateral pain were less accurate than those without pain. Those with bilateral pain and those with left-directional neck pain were both slower than those without neck pain. No significant interaction between direction of movement-induced neck pain and identifying a left-turning vs. right-turning neck/head image. Photo credit: Juliet Gore. [file peerj-08-8553-s002.pdf]

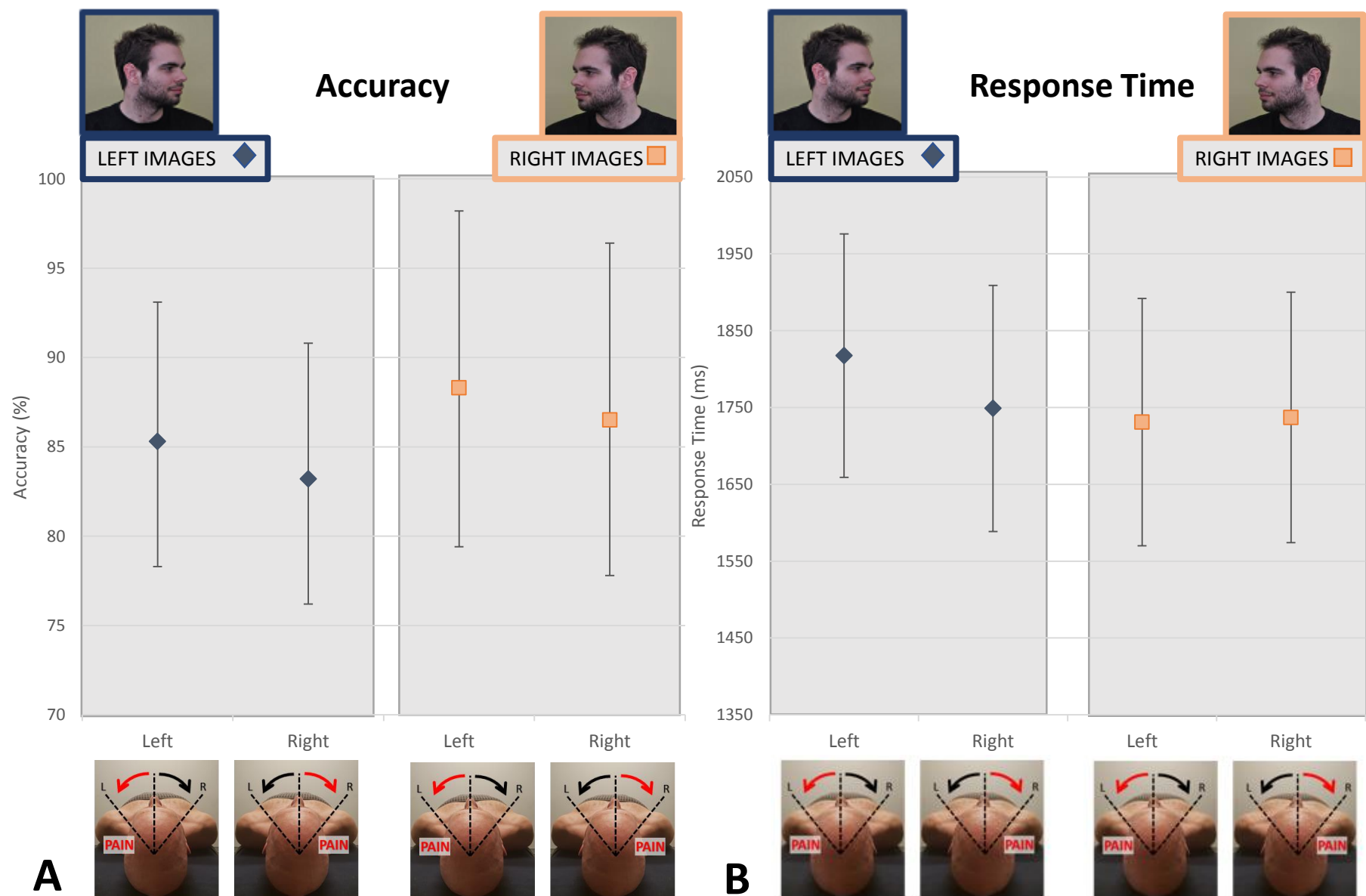

Direction of head turn associated with pain

Supplement: Supplemental Information 3 — (A) Accuracy; (B) Response time. No significant main effects were found. No significant interactions between movement-evoked pain location and ability to identify a right-turning vs. left-turning neck image. Photo credit: Juliet Gore. [file peerj-08-8553-s003.pdf]
